# Predicting AIDS-related events using CD4 percentage or CD4 absolute counts

**DOI:** 10.1186/1742-6405-3-20

**Published:** 2006-08-17

**Authors:** Yasmin Pirzada, Sadik Khuder, Haig Donabedian

**Affiliations:** 1 College of Medicine, University of Toledo, Department of Internal Medicine, 3120 Glendale Ave, Toledo, OH 43614, USA

## Abstract

**Background:**

The extent of immunosuppression and the probability of developing an AIDS-related complication in HIV-infected people is usually measured by the absolute number of CD4 positive T-cells. The percentage of CD4 positive cells is a more easily measured and less variable number. We analyzed sequential CD4 and CD8 numbers, percentages and ratios in 218 of our HIV infected patients to determine the most reliable predictor of an AIDS-related event.

**Results:**

The CD4 percentage was an unsurpassed predictor of the occurrence of AIDS-related events when all subsets of patients are considered. The CD4 absolute count was the next most reliable, followed by the ratio of CD4/CD8 percentages. The value of CD4 percentage over the CD4 absolute count was seen even after the introduction of highly effective HIV therapy.

**Conclusion:**

The CD4 percentage is unsurpassed as a parameter for predicting the onset of HIV-related diseases. The extra time and expense of measuring the CD4 absolute count may be unnecessary.

## Background

The great majority of clinical laboratories use single-platform flow cytometry which directly measures the percentage of lymphocytes which are CD4 positive [1]. In order to calculate the absolute CD4 T-cell cell count, a complete blood cell count must be done to determine the absolute white blood cell count. The absolute white blood cell number per microliter must then be multiplied by the fraction of white blood cells which are lymphocytes as determined by a manual differential count. The resulting lymphocyte count per microliter is multiplied by the measured CD4 percentage to yield an absolute CD4 count per microliter of blood. This method introduces two sources of measurement errors (WBC measurement and differential count). It also requires the drawing of blood into another tube as flow cytometry and CBC tubes require different anticoagulants. Pan-leukocyte gating with CD45 antibody is now standard procedure and it reduces the error in the CD4 determination, but it does not eliminate the variability inherent in the calculations.

In addition to extra blood drawn, extra work performed and the introduction of extra measurement errors, there is a problem with the variability of the absolute CD4 count throughout a 24 hour day in both normal and HIV-infected patients [2]. The Centers for Disease Control definition of AIDS allows the measurement of the CD4 percentage as an option for the diagnosis of AIDS [3] and as an option for instituting pneumocystis prophylaxis (but not for toxoplasma or MAI prophylaxis) [4]. Since the variance of the measurement of CD4 percentage is about one-half of that of the CD4 absolute count [5], utilization of CD4 percentage could be superior to reliance on the CD4 absolute count.

Masur *et al *[6] showed that the CD4 percentage is a valid predictor of the probability of developing pneumocystis pneumonia. A study of HIV positive Australian men without AIDS found that the time to develop AIDS could be predicted by the CD4 count, CD4 percentage and the rate of change for each [7]. This study excluded patients with AIDS and preceded the era of effective HIV therapy. A recently published study [8] showed that the CD4 percentage adds predictive value when disease progression is studied in HIV-infected people with more than 350 CD4 positive cells per microliter.

In order to better clarify the roles of the CD4 percentage and the CD4 absolute count in the care of HIV patients in the era of highly effective HIV therapy, we reviewed the clinical records of 218 of our HIV-infected patients to determine whether the CD4 percentage is superior to the CD4 absolute count in predicting the development of an HIV-related complication. If the CD4 percentage were superior or equivalent, unnecessary expense, time and measurement error could be prevented by measuring only the CD4 percentage.

## Methods

Two hundred and eighteen records of HIV-infected patients followed at the Medical University of Ohio were analyzed. The names of all HIV-infected patients seen at our institution were listed alphabetically and all retrievable charts which contained sufficient data for analysis were included. One hundred twenty-two patients were first seen after the advent of effective HIV therapy (after January 1, 1995). The remainder were first seen prior to January 1, 1995. We assume that all patients followed after January 1, 1995 were prescribed effective therapy as they were all seen by our infectious disease attending physicians; but we can not be certain that the patients were taking their therapy continuously or correctly. As such, the demographic expedient of a temporal divide was used.

All patients had a complete blood count as well as flow cytometry performed at times deemed appropriate by their physician. The flow cytometry was gated on light-scattering characteristics of the leukocytes and the identity of the lymphocytes confirmed by CD45 antibody positivity to exclude cellular debris.

The absolute lymphocyte count, CD4 absolute count, CD4 percentage, CD8 absolute count, the CD8 percentage and the CD4/CD8 ratio of the absolute counts and the ratio of the percentages were recorded at the first visit and on subsequent visits until an AIDS-related event occurred or until the most recent recorded laboratory result. There were 140 AIDS-related events among the 218 patients (Table 1).

**Table 1:** The incidence of AIDS-related events

Pneumocystis pneumonia	24
Candidal esophagitis	22
Cryptococcal meningitis or fungemia	11
AIDS wasting syndrome	9
Cytomegaloviral retinitis	9
Non-Hodgkin lymphoma	8
*Mycobacterium avium *bacteremia	8
Dementia or myelopathy	7
Kaposi sarcoma	6
Cerebral toxoplasmosis	5
Pancytopenia (RBC, WBC, Platelets)	5
Progressive multifocal leukoencephalopathy	5
Cytomegaloviral colitis	4
Tuberculosis	3
*M. avium *pneumonia	3
Cytomegaloviral pneumonia	2
Cardiomyopathy	2
Cytomegaloviral esophagitis	1
Microsporidial colitis	1
Cryptosporidial enteritis	1
*M. avium *colitis	1
Persistent herpes simplex	1
Aspergillosis	1
Disseminated cytomegalovirus infection	1
**Total Events**	**140**

Survival model methods were used to analyze the data. Before applying survival models, we compared the patient groups with and without AIDS-related events with regard to age and gender distribution. The number of months between the first clinic visit and the event or the end of the study was calculated. The median times to an AIDS-related event were calculated if appropriate and analyzed using the Cox regression model. We calculated R^2 ^as proposed by Cox and Snell [9] and used it to evaluate the usefulness of various measures in predicting the occurrence of an event. The R^2 ^statistic reflects the strength of association between the measured variable and the time to occurrence of an AIDS-associated event.

The number of months to an event were plotted against the CD4 count and the CD4 percentage for the time periods before and after 1995. A curve-fitting program was used to determine the best fit of a continuous curve to the data. The F statistic was used to compare pairs of fitted curves to determine which curve best fitted the plotted points.

## Results

Our patients' ages ranged from 21 to 72 and 83% were male. African Americans made up 34% of our population, Hispanics 4% and Caucasians 62%. These percentages reflect the prevalence of HIV infection in the USA. Nineteen percent of our male patients were infected via a heterosexual route and 6% were intravenous drug abusers. Only 3% our female patients were IVDU's. The number of measurements for each patient varied from 1 to 22, depending on the frequency of clinic visits and the rapidity of development of an AIDS-related illness.

We found that the absolute lymphocyte count was the best predictor of the time to an AIDS-related event (Table 2) with an R^2 ^of 0.887 and a p < 0.0001. The CD4 percentage was a close second with an R^2 ^of 0.857. The CD4 absolute count was of similar value with an R^2 ^of 0.813. The ratios of CD4 % to CD8 % and CD4 absolute to CD8 absolute counts were significantly predictive of the time to an event, but less so than the CD4 percentage alone. The CD8 percentage alone had the least predictive value.

**Table 2:** 

	**Chi-Square**	**R**^2^	**P-Value**
Absolute lymphocyte	44.3	0.887	<0.0001
CD4 percentage	42.1	0.857	<0.0001
CD4 absolute	38.9	0.813	<0.0001
CD8 percentage	3.7	0.015	0.0542
CD8 absolute	26.5	0.547	0.0001
CD4/CD8 percentage	35.8	0.761	<0.0001
CD4/CD8 absolute	33.6	0.720	<0.0001

The absolute lymphocyte count has been cited as a cost-effective means of following the progression of HIV disease, especially in areas where flow cytometry is unaffordable [10]. We were surprised at the power of the absolute lymphocyte count, but we did not pursue its use further since flow cytometry is universally accepted in the developed world as necessary to monitor the progression of HIV infection.

Since the survival of HIV infected patients improved greatly with the advent of more effective therapies after December 1994, we compared the predictive value of CD4 absolute and CD4 percentage values in patients first followed before 1995 with those first followed in 1995 or after (Table 3). Ninety-six patients were first seen before 1995 and 122 after January 1, 1995. When patients are segregated in this manner, the CD4 percentage is superior to the CD4 absolute count in patients in the highly effective therapy era (R^2 ^= 0.696). Note that the R^2 ^values are lower due to the fewer patients analyzed. Before 1995, the CD4 absolute count has slightly more predictive value than the CD4 percentage (R^2 ^= 0.550 vs 0.513).

**Table 3:** 

	**Chi-Square**	**R**^2^	**P-Value**
**Before 1995**			
CD4 percentage	16.2	0.513	<0.0001
CD4 absolute	16.9	0.550	<0.0001
**After 1995**			
CD4 percentage	25.5	0.696	<0.0001
CD4 absolute	22.2	0.596	<0.0001

We also analyzed the predictive value of the CD4 absolute count and percentage when the CD4 count was stratified as less than 201 and 201 to 350 at the first measurement. These data (Table 4) show that the CD4 percentage remains the best predictor of an adverse event when the CD4 count is between 201 and 350, but when the count drops below 201, the CD4 absolute count has more predictive value.

**Table 4:** 

	**Chi-Square**	**R**^2^	**P-Value**
**CD4 201–350**			
CD4 percentage	2.94	0.05	0.087
CD4 absolute	0.15	0.0001	0.703
**CD4 <201**			
CD4 percentage	9.84	0.193	0.002
CD4 absolute	17.44	0.50	<0.0001

**Figure 1:**
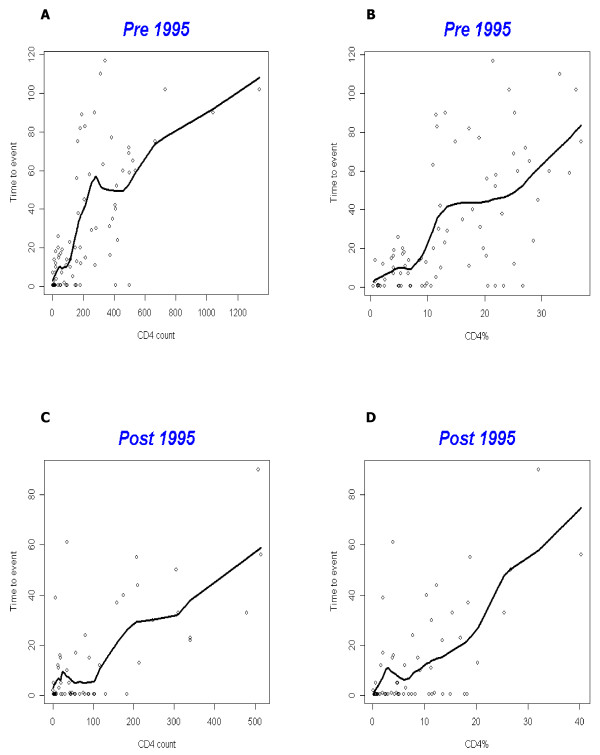
The time to event in months is plotted against the CD4 absolute (A and C) or the CD4 percentage (B and D). The patients who initially presented with an AIDS-related event were censored. The curves were plotted for patients initially seen before 1995 (A and B) and after 1995 (C and D).

In order to examine the time to an event, not just its occurrence, we plotted the CD4 count and the CD4 percentage against the time to an event. This was done for patients first seen prior to 1995 and for those first seen after 1995 (Figure 1). Those patients who presented with an AIDS-related event were censored from the curves. The computer-fitted curves are not monotonic, but display the expected relationship of increasing time to event with increasing CD4 absolute count or CD4 percentage. For both pre and post 1995 sets of curves, the CD4 percentage more accurately predicts the time to an event than does the CD4 absolute count. The F-statistic is a measure of relative goodness of fit between two curves and the p values of the F statistic are p = 0.0002 and 0.0006 in favor of the CD4 percentage for both groups of patients.

## Discussion

In spite of several studies over the last twenty years, the relative value of the CD4 percentage and CD4 absolute count in predicting the onset of AIDS-related events is not settled. A recently published study by Gebo *et al *of Johns Hopkins patients [11] attempts to resolve this issue using an analysis of repeated binary variables. This study found that for any CD4 percentage quartile, the CD4 absolute count has additional predictive value. Our study looked at the time interval between each measurement and the occurrence of the first AIDS-related event, whereas the Gebo study looked at the presence or absence of multiple events for up to 6 months after the laboratory measurement. We believe our time to event analysis has more relevance to the clinician and the patient since it informs them about the expected time interval in which to intervene. This is important in counseling patients who may have concerns about the negative aspects of HIV therapy and want to know how long they can wait before instituting therapy. With the exception of patients who have a CD4 absolute count below 200 and thus have AIDS by the Centers for Disease Control's definition [12], the CD4 percentage is superior or equivalent to the CD4 absolute count in various subsets of HIV infected patients. If only patients who meet the CDC definition of AIDS are analyzed (CD4 count <200), the CD4 absolute count is a better predictor of the true to onset of an event. This may be due to the absolute lymphopenia seen in very ill AIDS patients which is best reflected in the absolute count.

A recent French study of HIV therapy-naïve patients between 1996–2002 used a survival analysis to determine the relative value of the CD4 percentage and count in describing the probability of an AIDS-related event or death. [13] They could not show any difference between the CD4 percentage or count in their survival curves.

We believe that the surprising power of the absolute lymphocyte count in predicting the time to an AIDS-related event stems both from the lymphopenia seen in malnutrition associated with advanced AIDS as well as the depletion of the CD4 positive population. Since the management of AIDS is dependent on CD4 enumeration, we do not advocate the use of the absolute lymphocyte count as a predictor of AIDS-related events.

We analyzed our data graphically in Figure 1 in order to show the distribution of the time to event for any given CD4 measurement. Figure 1 is divided into pre and post 1995 patients to give a more relevant analysis for the present effective therapy era. As expected, there is a considerable scatter in the number of months to an event for any given CD4 measurement, but the pairs of curves for CD4 percentage and CD4 absolute count are similar in shape when compared for both time periods. The F statistic shows that the fit is better in both cases when the CD4 percentage is plotted against the time to event.

The average slopes of the pre-1995 curves are steeper than the post-1995 curves. This is most evident when the CD4 absolute curves are compared (1A and 1C). Why is an event more likely for a given CD4 value after 1995? Since these curves record only those patients who attained an event endpoint, the post-1995 curves represent those patients who probably were not compliant with their medication and who may have engaged in activities which increased their probability of reaching an event. The pre-1995 curves include patients who were taking no medication or relatively ineffective medication. Compliance issues were therefore less important and the pre-1995 curves probably reflect a broader cross-section of our patients.

## Conclusion

The time interval between laboratory testing and the development of an AIDS-related disease is adequately predicted by the CD4 percentage when our 218 patients are taken as a whole. When different subsets are examined, the CD4 percentage is better or equal to the CD4 absolute count with the exception of those with a CD4 count below 201. In that subset the CD4 absolute count is the best predictor, but the CD4 percentage is still an accurate predictor.

We conclude that the CD4 percentage may be used instead of the CD4 count to predict the time to an AIDS-related event. The extra work and expense of the CD4 absolute count determination may not be necessary.

## Abbreviations

CD Clusters of differentiation

HIV Human immunodeficiency virus

## Competing interests

The author(s) declare that they have no competing interests.

## Authors' contributions

All authors have read and approved the final manuscript.
